# A nonparametric Bayesian continual reassessment method in single-agent dose-finding studies

**DOI:** 10.1186/s12874-018-0604-9

**Published:** 2018-12-18

**Authors:** Niansheng Tang, Songjian Wang, Gen Ye

**Affiliations:** Key Lab of Statistical Modeling and Data Analysis of Yunnan Province, Yunnan University, Kunming, 650091 People’s Republic of China

**Keywords:** Adaptive rejection Metropolis sampling algorithm, Continual reassessment method, Dirichlet process prior, Dose-finding design, Gibbs sampler, Maximum tolerated dose

## Abstract

**Background:**

The main purpose of dose-finding studies in Phase I trial is to estimate maximum tolerated dose (MTD), which is the maximum test dose that can be assigned with an acceptable level of toxicity. Existing methods developed for single-agent dose-finding assume that the dose-toxicity relationship follows a specific parametric potency curve. This assumption may lead to bias and unsafe dose escalations due to the misspecification of parametric curve.

**Methods:**

This paper relaxes the parametric assumption of dose-toxicity relationship by imposing a Dirichlet process prior on unknown dose-toxicity curve. A hybrid algorithm combining the Gibbs sampler and adaptive rejection Metropolis sampling (ARMS) algorithm is developed to estimate the dose-toxicity curve, and a two-stage Bayesian nonparametric adaptive design is presented to estimate MTD.

**Results:**

For comparison, we consider two classical continual reassessment methods (CRMs) (i.e., logistic and power models). Numerical results show the flexibility of the proposed method for single-agent dose-finding trials, and the proposed method behaves better than two classical CRMs under our considered scenarios.

**Conclusions:**

The proposed dose-finding procedure is model-free and robust, and behaves satisfactorily even in small sample cases.

## Background

Dose-finding designs for phase I trials have been widely discussed over the past two decades. Many methods have been proposed to identify maximum tolerated dose (MTD) in single-agent dose-finding clinical trials. There are two major branches: model-based and algorithm-based methods. Among the model-based methods, the continual reassessment method (CRM) proposed by O’Quigley et al. [1] is a quite popular dose-finding approach. Since O’Quigley et al.’s pioneer work, there is considerable literature on modifying or improving CRM. For example, see Whitehead and Brunier [2] for Bayesian decision-theoretic approach, Piantadosi et al. [3] for a modified CRM, Heyd and Carlin [4] for an adaptive design improvement of the CRM, Leung and Wang [5] for a decision-theory-based extension of the CRM, Yuan et al. [6] for the quasi-likelihood approach, Yin and Yuan [7] for Bayesian model averaging CRM, Møller [8] for the up-and-down design, Fan et al. [9] for a simple Bayesian decision-theoretic design. Recently, Morita et al. [10] extended the CRM to a hierarchical Bayesian dose-toxicity model that borrows strength between subgroups under the assumption that the subgroups are exchangeable. However, in many practical applications, the true dose-toxicity curve is unknown, thus specifying an explicit dose-toxicity curve via some parametric model is artificial and may largely increase complications.

The algorithm-based approach, which can be regarded as a “nonparametric” or model-free method, has received considerable attention over the past years. There is considerable literature on model-free methods from a Bayesian nonparametric viewpoint. For example, Gelfand and Kuo [11] developed a fully Bayesian nonparametric approach to estimate the dose-toxicity curve with the Dirichlet process (DP) prior and the product-of-beta prior in the quantal bioassay problem. Mukhopadhyay [12] adopted the DP prior to make inference on the dose level with a prespecified response rate. Gasparini and Eisele [13] developed a curve-free method to improve CRM by modeling toxicity probabilities with the product-of-beta prior. The product-of-beta prior is conjugate with the binomial distribution, but it has a well-known numerical problem for exact computation and involves many hyperparameters to be determined, which may affect the performance of the considered method [14]. Ivanova and Wang [15] developed a nonparametric approach to estimate MTD for each of two strata of patients. Yan et al. [16] presented a Bayesian design for dose escalation and de-escalation. To our knowledge, no literature exists on Bayesian nonparametric dose-finding approach in single-agent clinical trials using the Dirichlet process prior.

The main purpose of this paper is to develop a flexible nonparametric statistical model to estimate unknown dose-toxicity curve for single-agent phase I clinical trials from a Bayesian viewpoint. This is implemented by treating toxicity probabilities as unknown parameters of interest which is subject only to monotonicity assumption, which is equivalent to assuming that the dose-toxicity curve is an arbitrary right continuous nondecreasing function whose range lies in [0,1], and then specifying a Dirichlet process prior for the unknown dose-toxicity curve. Also, a two-stage Bayesian nonparametric adaptive dose-finding design is developed to estimate MTD in single-agent dose-finding clinical trials. The main merits of the proposed method include that (i) the assumption of a specific dose-toxicity curve in CRM is not necessary; (ii) there are only two hyperparameters in the specified DP prior; (iii) there is more information that can be used to estimate toxicity probabilities and MTD; (iv) the escalation or deescalation of the current dose to the adjacent dose is only implemented once, thus the highest toxicity dose can be adaptively reached; (v) doses with relatively high toxicity probability may have no chance to be assigned to patients, which guarantees the safety of patients.

The rest of this paper is organized as follows. In “Methods” section, we briefly review the traditional CRM in single-agent dose-finding clinical trials, and introduce Bayesian nonparametric probability model by imposing a DP prior on the unknown dose-toxicity curve. A hybrid algorithm combining the Gibbs sampler and adaptive rejection Metropolis sampling (ARMS) algorithm is developed to estimate toxicity probabilities in “Methods” section. In “Dose-finding algorithm” section, a two-stage Bayesian nonparametric adaptive dose-finding algorithm is presented to estimate MTD in single-agent dose-finding clinical trials. Simulation studies are conducted to investigate the finite sample performance of the proposed method in “Simulation study” section. Some concluding remarks are given in “Results” section.

## Methods

Let *N* be the total number of patients, *J* be the total number of cohorts, *m* be the number of patients in each of *J* cohorts. Let *d*_1_<*d*_2_<⋯<*d*_*K*_ be a set of *K* pre-specified dose levels for a single agent under investigation, *θ* be the target toxicity probability, the dose-toxicity curve *F*(·) be the distribution of dose levels, *p*_*k*_=*F*(*d*_*k*_) be the toxicity probability at dose level *d*_*k*_ for *k*=1,…,*K*. It is assumed that there are *j* cohorts enrolled in the current experiment. Let $\boldmath {n}^{j}=\left (n_{1}^{j},\ldots,n_{K}^{j}\right)^{\!\top \!}$ and $\boldmath {y}^{j}=\left (y_{1}^{j},\ldots,y_{K}^{j}\right)^{\!\top \!}$, where $n_{k}^{j}$ is the number of patients treated at dose level *d*_*k*_ in the first *j* cohorts, and $y_{k}^{j}$ represents the number of patients experienced the dose-limiting toxicity (DLT) among $n_{k}^{j}$ patients treated at dose level *d*_*k*_. Let *S*_*j*_ be the number of non-zero components in *n*^*j*^. Thus, we have *S*_*j*_≤*K*. That is, only doses $d_{1},\ldots,d_{\phantom {\dot {i}\!}S_{j}}$ are administered to the first *j* cohorts until now. Let $D_{\phantom {\dot {i}\!}S_{j}}=\left \{\left (n_{1}^{j},y_{1}^{j}\right),\ldots, \left (n_{\phantom {\dot {i}\!}S_{j}}^{j},y_{\phantom {\dot {i}\!}S_{j}}^{j}\right)\right \}$ be the data collected from the first *j* cohorts of patients, $\boldmath {p}=(p_{1},\ldots,p_{\phantom {\dot {i}\!}S_{j}})^{\!\top \!}$ be a set of toxicity probabilities corresponding to $\{d_{1},\ldots,d_{\phantom {\dot {i}\!}S_{j}}\}$. For simplicity, in what follows, we omit superscript *j* in *n*^*j*^ and *y*^*j*^.

### The continual reassessment method (CRM)

Let *p*_01_<…<*p*_0*K*_ be an initial guess of toxicity probabilities associated with dose levels *d*_1_<…<*d*_*K*_, where *p*_0*k*_ is referred to as the “skeleton” of the CRM. It is assumed that there is a prior belief that dose level *d*_*k*_ is the MTD, one may set the initial guess as *p*_0*k*_=*θ*. In practice, clinical investigators attempt to provide their best guess on the “skeleton”. However, in simulation study, the “skeleton” can be directly generated using the function ‘ *getprior*(*δ,θ,k,K*)’ in the R package ‘dfcrm’, where *δ* is the desired length of the indifference interval and the optimal value of *δ* can be calibrated by the algorithm given in Lee and Cheung [17], *θ* is the target toxicity probability, *k* is the location of the target dose level and *K* is the total number of dose levels. More discussions on the guess of the “skeleton” of the CRM see [17]. In the Bayesian framework, the CRM assumes that the dose-toxicity relationship *F*(*d*_*k*_) is a strictly increasing function with respect to the dose level *d*_*k*_, and is usually specified by a parametric model *F*(*d*_*k*_;*β*) in which *β* is an unknown parameter to be estimated, such as, logistic function, power function, and hyperbolic function.

Let *x*_*i*_∈{*d*_1_,…,*d*_*K*_} be the dose assigned to the *i*th cohort for *i*=1,…,*j*. Suppose that *y*_*i*_ patients have experienced DLT among *n*_*i*_ patients treated at dose level *x*_*i*_. Thus, *y*_*i*_ follows the binomial distribution with the toxicity probability *F*(*x*_*i*_;*β*). Let *D*_*j*_={*y*_1_,…,*y*_*j*_} be the dataset collected from the first *j* cohorts of patients. The likelihood function for *D*_*j*_ is given by

$$L_{j}(\beta|D_{j})\propto\prod_{i=1}^{j}\{F(x_{i};\beta)\}^{\phantom{\dot{i}\!}y_{i}}\{1-F(x_{i};\beta)\}^{\phantom{\dot{i}\!}n_{i}-y_{i}}. $$

Let *f*(*β*) be the prior density of *β*. It follows from the Bayesian theorem that a Bayesian estimator of *β* is given by

$$\hat{\beta}_{j}=E_{\phantom{\dot{i}\!}\beta|D_{j}}(\beta)=\frac{\int\beta L_{j}(\beta|D_{j})f(\beta)d\beta}{\int L_{j}(\beta|D_{j})f(\beta)d\beta}, $$

where $E_{\phantom {\dot {i}\!}\beta |D_{j}}$ is the expectation taken with respect to the posterior probability density function $f\left (\beta |D_{j}\right)=L_{j}(\beta |D_{j})f(\beta)/\int L_{j}(\beta |D_{j})f(\beta)d\beta $. The (*j*+1)th cohort of patients is assigned to the dose level

$$x_{j+1}=\text{arg}\min_{\phantom{\dot{i}\!}d_{k}}|F\left(d_{k};\hat{\beta}_{j}\right)-\theta|, ~k=1,\ldots,K, $$

where *θ* is the target toxicity probability. Once the maximum sample size *N* is reached, the dose with the toxicity probability closest to *θ* is selected as the MTD.

### Nonparametric probability model

The model-based approaches such as CRM and the modified CRMs are widely used in single-agent dose-finding clinical trials. However, parametric modeling of *F*(·) may be problematical when there is little prior information on the shape of the dose-toxicity curve [12]. Moreover, in some experimental situations, it is recognized that parametric models do not work [18]. To address these issues, we assume that *F*(·) is an arbitrary right continuous nondecreasing unknown function whose range is [0,1], and can be approximated using some nonparametric method. To this end, a Dirichlet process (DP) prior is adopted to approximate *F*(·). To wit, we assume *F*(*d*_*k*_)∼DP(*αF*_0_(*x*|*η*)), where *F*_0_(*x*|*η*) is a base distribution that serves as a starting point for constructing a nonparametric distribution for *F*(*d*_*k*_), *η* is a parameter associated with the base distribution, *α* represents the weight that a researcher assigns a priori to the base distribution, and reflects the closeness of *F*_0_(*x*|*η*) to *F*(*d*_*k*_). Thus, the hierarchical model for the dataset {*y*_1_,…,*y*_*K*_} is given by

1 $$ \begin{array}{lll} y_{k}|p_{k} \overset{\text{ind}}{\sim}{\text{Bin}(n_{k},p_{k}),\ \ k=1,\ldots,K,}\\ p_{k}=F(d_{k}),\ \ \ F|\eta,\alpha \sim \text{DP}(\alpha F_{0}(x|\eta)). \end{array}  $$

In many applications, *α* is usually unknown. Many methods have been proposed to select the prior of *α* (e.g., see [19, 20]). Here, we assume that the prior distribution of *α* follows a Gamma distribution with hyperparameters *a* and *b*, i.e., *α*∼*Γ*(*a,b*). The base distribution *F*_0_(*x*|*η*) is a prior guess of the unknown distribution *F*(·). For computational simplicity, *F*_0_(*x*|*η*) should be a conjugate prior, such as a uniform distribution or a mixture of two types of distributions. Also, one may consider empirical Bayesian and noninformative prior for *F*_0_(*x*|*η*). More discussions on the selection of *F*_0_(·) see Mukhopadhyay [12]. Here, we take *F*_0_ as the cumulative distribution function of some standard distribution, and choose some appropriate hyperparameters such that the median of *F*_0_ should be consistent with the initial guess for the MTD. For example, if there is a prior belief that dose level *d*_*k*_ is the MTD, we can select appropriate values of *μ* and *σ* such that $F_{0}(d_{k}|\eta)=\Phi \left (\frac {d_{k}-\mu }{\sigma }\right)=\theta $, where *η*={*μ,σ*}, *μ* is associated with the mean of *F*_0_, and *σ* is related with the standard variance of *F*_0_. In this case, similar to [12], we assume that the priors of *μ* and *σ* discretely follow the uniform distribution in the intervals [*μ*_0_−*r,μ*_0_+*r*] (e.g., taking ten equally spaced points in [*μ*_0_−*r,μ*_0_+*r*]) and [*σ*_0_−*r,σ*_0_+*r*] (e.g., taking ten equally spaced points in [*σ*_0_−*r,σ*_0_+*r*]) with *r*∈{1,2}, respectively, where *μ*_0_ and *σ*_0_ are the mean and standard variance of *F*_0_, respectively.

According to the definitions of *μ* and *σ*, it is logical to assume that the joint prior density of *μ* and *σ* has the form of *π*(*μ,σ*)=*π*(*μ*)*π*(*σ*). Thus, the hierarchical model can be written as

2 $$ \begin{array}{lll} y_{k}|p_{k} \overset{\text{ind}}{\sim} \text{Bin}(n_{k},p_{k}),\ \ k=1,\ldots,K,\\ p_{k}=F(d_{k}), \ \ \ F|\eta,\alpha \sim \text{DP}(\alpha F_{0}(x|\eta)),\\ \alpha \sim \Gamma(a,b), \ \ \ \eta \sim \pi(\mu)\pi(\sigma). \end{array}  $$

By the definition of the DP prior, for any finite measurable partition *B*_1_,…,*B*_*K*_ in the support of *F*_0_, the probability vector (*F*(*B*_1_),…,*F*(*B*_*K*_))^⊤^ follows the Dirichlet distribution with parameter vector (*αF*_0_(*B*_1_),…, *αF*_0_(*B*_*K*_))^⊤^. For the base distribution *F*_0_(*x*|*η*)=*N*(*x*;*μ,σ*), if we consider the following finite measurable partition for the support (−*∞,∞*): *B*_1_=(−*∞,d*_1_), *B*_2_=[*d*_1_,*d*_2_),…,*B*_*K*_=[*d*_*K*−1_,*d*_*K*_), *B*_*K*+1_=[*d*_*K*_,+*∞*), thus we have *αF*_0_(*B*_1_)=*αF*_0_(*d*_1_),…,*αF*_0_(*B*_*K*+1_)=*α*[1−*F*_0_(*d*_*K*_)], and *F*(*B*_1_)=*p*_1_, *F*(*B*_2_)=*p*_2_−*p*_1_,…,*F*(*B*_*K*+1_)=1−*p*_*K*_. Then, the joint prior density *π*(*p*) of *p*=(*p*_1_,…,*p*_*K*_)^⊤^ can be written as

3 $$ \pi(\boldmath{p})=\frac{\Gamma\left(\sum\limits_{k=1}^{K+1}\gamma_{k}\right)}{\prod\limits_{k=1}^{K+1} \Gamma(\gamma_{k})}\prod\limits_{k=1}^{K+1}(p_{k}-p_{k-1})^{\phantom{\dot{i}\!}\gamma_{k}-1},  $$

where *γ*_*k*_=*α*{*F*_0_(*d*_*k*_)−*F*_0_(*d*_*k*−1_)} for *k*=1,…,*K*+1, *d*_0_=−*∞*, *d*_*K*+1_=*∞*, *F*_0_(−*∞*)=0, *F*_0_(*∞*)=1, *p*_0_=0, and *p*_*K*+1_=1.

### Conditional distributions

Under the above assumption, the likelihood function of the first *j* (*j*=1,…,*J*) cohorts of patients (i.e., the dataset $D_{\phantom {\dot {i}\!}S_{j}}=\{(n_{1},y_{1}),\ldots, (n_{\phantom {\dot {i}\!}S_{j}},y_{\phantom {\dot {i}\!}S_{j}})\}$) is given by

4 $$ L_{\phantom{\dot{i}\!}S_{j}}(\boldmath{p}|D_{\phantom{\dot{i}\!}S_{j}})\propto\prod\limits_{i=1}^{S_{j}} p_{i}^{\phantom{\dot{i}\!}y_{i}}(1-p_{i})^{\phantom{\dot{i}\!}n_{i}-y_{i}},~1\leq S_{j}\leq K.  $$

It follows from Eq. (3) that the joint prior density *π*(*p*) of $\boldmath {p}=(p_{1},\ldots,p_{\phantom {\dot {i}\!}S_{j}})^{\!\top \!}$ can be expressed as

5 $$  \pi(\boldmath{p}) \propto \prod\limits_{i=1}^{S_{j}+1}(p_{i}-p_{i-1})^{\phantom{\dot{i}\!}\gamma_{i}-1},\ \ 1\leq S_{j}\leq K,  $$

where *γ*_*i*_=*α*{*F*_0_(*d*_*i*_)−*F*_0_(*d*_*i*−1_)} for *i*=1,…,*S*_*j*_+1, *d*_0_=−*∞*, $d_{\phantom {\dot {i}\!}S_{j}+1}=\infty $, *F*_0_(−*∞*)=0, *F*_0_(*∞*)=1, *p*_0_=0, and $p_{\phantom {\dot {i}\!}S_{j}+1}=1$. It is easily seen that Eq. (5) reduces to Eq. (3) when *S*_*j*_=*K*.

It follows from Eqs. (4) and (5) that the joint posterior probability density of *p* given the dataset $D_{\phantom {\dot {i}\!}S_{j}}$ can be expressed as

6 $$ \pi(\boldmath{p}|D_{\phantom{\dot{i}\!}S_{j}})\!\propto\! \prod\limits_{i=1}^{S_{j}} p_{i}^{\phantom{\dot{i}\!}y_{i}}(1-p_{i})^{\phantom{\dot{i}\!}n_{i}-y_{i}}\!\prod\limits_{i=1}^{S_{j}+1}\!\!(p_{i}-p_{i-1})^{\phantom{\dot{i}\!}\gamma_{i}-1},\ \ \!\!1\!\leq\! S_{j}\!\leq \!K.  $$

Again, it follows from Eqs. (2) and (3) that the joint conditional distribution of {*α,μ,σ*} given $\{D_{\phantom {\dot {i}\!}S_{j}},\boldmath {p}\}$ is given by

$${}\pi(\alpha,\mu,\sigma|\boldmath{p},D_{\phantom{\dot{i}\!}S_{j}})\propto \frac{\Gamma\left(\sum\limits_{i=1}^{S_{j}+1}\gamma_{i}\right)}{\prod\limits_{i=1}^{S_{j}+1} \Gamma(\gamma_{i})}\prod_{i=1}^{S_{j}+1}(p_{i}-p_{i-1})^{\phantom{\dot{i}\!}\gamma_{i}-1}\pi(\alpha)\pi(\mu)\pi(\sigma), $$

which indicates that the conditional distributions of *α*, *μ* and *σ* given $\{\boldmath {p},D_{\phantom {\dot {i}\!}S_{j}}\}$ have the following expressions:

7 $$ \begin{array}{lll} \pi(\alpha|\boldmath{p},\mu,\sigma,D_{\phantom{\dot{i}\!}S_{j}}) &\propto& \frac{\Gamma(\alpha)}{\prod\limits_{i=1}^{S_{j}+1}\Gamma(\gamma_{i})}\prod\limits_{i=1}^{S_{j}+1}(p_{i}-p_{i-1})^{\phantom{\dot{i}\!}\gamma_{i}-1}\alpha^{a-1}e^{-b\alpha}, \\ \pi(\mu,\sigma|\boldmath{p},\alpha,D_{\phantom{\dot{i}\!}S_{j}}) &\propto & \frac{\Gamma(\alpha)}{\prod\limits_{i=1}^{S_{j}+1}\Gamma(\gamma_{i})}\prod\limits_{i=1}^{S_{j}+1}(p_{i}-p_{i-1})^{\phantom{\dot{i}\!}\gamma_{i}-1}\pi(\mu)\pi(\sigma).\\ \end{array}  $$

It is easily seen from Eqs. (6) and (7) that the conditional distributions of *p* and *α* are not standard distributions. Thus, it is quite difficult to draw observations from these conditional distributions. To address the issue, the Gibbs sampler is employed to simulate observations from these conditional distributions. Thus, Bayesian estimate of *p* can be obtained by the simulated observations via the Gibbs sampler.

### Implementation of Gibbs sampler

Let *p*_−*i*_ be a subset vector of *p* with the *i*th element of *p* deleted. It follows from Eq. (6) that the conditional distribution of *p*_*i*_ given *p*_−*i*_ and $D_{\phantom {\dot {i}\!}S_{j}}$ can be written as

8 $$ {}\pi(p_{i}|\boldmath{p}_{-i},D_{\phantom{\dot{i}\!}S_{j}})\!\propto p_{i}^{\phantom{\dot{i}\!}y_{i}}\!(1\!-p_{i})^{\phantom{\dot{i}\!}n_{i}-y_{i}}\!(p_{i}-p_{i-1})^{\phantom{\dot{i}\!}\gamma_{i}-\!1}\!(p_{i+1}\,-\,p_{i})^{\phantom{\dot{i}\!}\gamma_{i+1}-1},  $$

where *p*_*i*−1_<*p*_*i*_<*p*_*i*+1_ for *i*=1,…,*S*_*j*_, and *p*_0_=0 and $p_{\phantom {\dot {i}\!}S_{j}+1}=1$.

The Gibbs sampler for drawing observations $\alpha,\mu,\sigma,p_{1},\ldots, p_{\phantom {\dot {i}\!}S_{j}}$ is implemented as follows.

**Step 1**. Initialize *α*^(0)^,*μ*^(0)^,*σ*^(0)^, $\boldmath {p}^{(0)}=\left (p_{1}^{(0)},p_{2}^{(0)},\ldots,\right. \left.p_{\phantom {\dot {i}\!}S_{j}}^{(0)}\right)$, and let *p*_0_=0 and $p_{\phantom {\dot {i}\!}S_{j}+1}=1$.

**Step 2**. At the (*q*+1)th iteration with current values $\alpha ^{(q)}, \mu ^{(q)}, \sigma ^{(q)}, \boldmath {p}^{(q)}=\left (p_{1}^{(q)},p_{2}^{(q)}, \ldots,p_{\phantom {\dot {i}\!}S_{j}}^{(q)}\right)$, generate *α*^∗^ from the uniform distribution *U*(1,20) and *u* from the uniform distribution *U*(0,1), respectively, if $u\leq \min \!\left \{\!1, \pi \!\!\left (\alpha ^{*}|\boldmath {p}^{(q)},\!\mu ^{(q)}, \!\sigma ^{(q)},\!D_{\phantom {\dot {i}\!}S_{j}}\right)\!/\pi \!\left (\alpha ^{(q)}|\boldmath {p}^{(q)},\!\mu ^{(q)},\sigma ^{(q)},\!D_{\phantom {\dot {i}\!}S_{j}}\right)\!\right \}$, we let *α*^(*q*+1)^=*α*^∗^; otherwise, we let *α*^(*q*+1)^=*α*^(*q*)^.

**Step 3**. Generate *μ*^(*q*+1)^ from the conditional distribution $\pi \left (\mu |\boldmath {p}^{(q)},\alpha ^{(q)}, \sigma ^{(q)}, D_{\phantom {\dot {i}\!}S_{j}}\right)$;

**Step 4**. Generate *σ*^(*q*+1)^ from the conditional distribution $\pi \left (\sigma |\boldmath {p}^{(q)},\alpha ^{(q+1)}, \mu ^{(q+1)},D_{\phantom {\dot {i}\!}S_{j}}\right)$;

**Step 5**. Simulate $p_{1}^{(q+1)}$ from the conditional distribution: $\pi _{\phantom {\dot {i}\!}S_{j}}\left (p_{1}|\alpha ^{(q+1)},\mu ^{(q+1)}, \sigma ^{(q+1)}, p_{2}^{(q)},\ldots,p_{\phantom {\dot {i}\!}S_{j}}^{(q)},D_{\phantom {\dot {i}\!}S_{j}}\right)$.

**Step 6**. Generate $p_{2}^{(q+1)}$ from the conditional distribution:

9 $$ {}\pi_{\phantom{\dot{i}\!}S_{j}}\!\!\left(p_{2}|\alpha^{(q+1)}, \mu^{\!(q+1)},\!\sigma^{\!(q+1)}, \!p_{1}^{(q+1)},p_{3}^{(q)},\ldots,p_{\phantom{\dot{i}\!}S_{j}}^{(q)},\!p_{\phantom{\dot{i}\!}S_{j}+\!1},D_{\phantom{\dot{i}\!}S_{j}}\!\right)\!.  $$

$$\hspace{3.5cm}\vdots$$

**Step*S***_***j***_**+4**. Draw $p_{\phantom {\dot {i}\!}S_{j}}^{(q+1)}$ from the conditional distribution: $\pi _{\phantom {\dot {i}\!}S_{j}}\!\left (p_{\phantom {\dot {i}\!}S_{j}}|\alpha ^{(q+1)}, \!\mu ^{(q+1)},\!\sigma ^{(q+1)}, \!p_{1}^{(q+1)},\!p_{2}^{(q+1)},\ldots, \!p_{\phantom {\dot {i}\!}S_{j}-1}^{(q+1)},D_{\phantom {\dot {i}\!}S_{j}}\right)$.

**Step*S***_***j***_**+5**. Repeat Step 2 to Step *S*_*j*_+4 until the convergence of the algorithm.

It is easily seen from Eq. (8) that it is impossible to directly draw observations from the conditional distribution of *p*_*i*_ given $(\boldmath {p}_{-i},D_{\phantom {\dot {i}\!}S_{j}})$ because of nonstandard distribution involved. To address the issue, the Adaptive Rejection Metropolis Sampling (ARMS) is employed to sample observations from the conditional distribution (8).

To implement the ARMS algorithm, we require constructing a proposal distribution from which one can easily sample observations. Let $D_{\phantom {\dot {i}\!}n_{0}}=\{x_{1}< x_{2}<\cdots < x_{\phantom {\dot {i}\!}n_{0}}\}$ denote a set of dose levels in the interval [*p*_*i*−1_,*p*_*i*+1_]. For 1≤*i*≤*j*≤*n*_0_, let *l*_*ij*_(*x*) be the straight line passing through two points (*x*_*i*_, log*π*(*x*_*i*_)) and (*x*_*j*_, log*π*(*x*_*j*_)); otherwise, it is assumed that *l*_*ij*_(*x*) is not defined. We construct the following piecewise function *h*_*i*_(*x*):

10 $$\begin{array}{*{20}l} h_{i}(x)=&\max\{l_{i,i+1}(x),\min\{l_{i-1,i}(x),l_{i+1,i+2}(x)\}\}\\ &\;\text{for}\ \ x_{i}\leq x\leq x_{i+1}. \end{array} $$

When *l*_1_(*x*) is undefined but *l*_2_(*x*) is defined above, we set max(*l*_1_,*l*_2_)= max(*l*_2_,*l*_1_)= min(*l*_1_,*l*_2_)= min(*l*_2_,*l*_1_)=*l*_2_. When both *l*_1_(*x*) and *l*_2_(*x*) are undefined, we take max(*l*_1_,*l*_2_)= max(*l*_2_,*l*_1_)= min(*l*_1_,*l*_2_)= min(*l*_2_,*l*_1_)=0. Under the above assumption, the proposal distribution *g*_*i*_(*x*) is defined as *g*_*i*_(*x*)= exp(*h*_*i*_(*x*))/*ω*, which is a piecewise exponential distribution, where $\omega =\int \exp (h(x))dx$. Let $p_{i}^{(q)}$ be the simulated observation of *p*_*i*_ at the *q*th iteration. Then, the ARMS algorithm for drawing $p_{i}^{(q+1)}$ from the conditional distribution (8) at the (*q*+1)th iteration is as follows.

**Step 1**. Initialize *n*_0_, which is the number of points in the interval [*p*_*i*−1_,*p*_*i*+1_], and determine the set of dose levels $D_{\phantom {\dot {i}\!}n_{0}}$.

**Step 2**. Generate $p_{i}^{*}$ from the proposal distribution *g*_*i*_(*x*).

**Step 3**. Generate *u* from the uniform distribution *U*(0,1).

**Step 4**. If $u>\pi (p_{i}^{*})/\exp (h_{i}(p_{i}^{*}))$, we set $D_{\phantom {\dot {i}\!}n_{0}+1}=D_{\phantom {\dot {i}\!}n_{0}}\cup \left \{p_{i}^{*}\right \}$, and relabel the points in $D_{\phantom {\dot {i}\!}n_{0}+1}$ in ascending order and let *n*_0_=*n*_0_+1, then go to step 2; otherwise, we set $p_{i}^{*(q+1)}=p_{i}^{*}$.

**Step 5**. Generate *u*^∗^ from the uniform distribution *U*(0,1).

**Step 6**. If $u^{*}\!>\!\min \!\left \{\!\!1,\!\frac {\pi \!\left (p_{i}^{*(q+1)}\right)\min \left \{\pi \left (p_{i}^{(q)}\!\right),\, \exp \left (h\left (p_{i}^{(q)}\right)\!\right)\right \}}{\pi \!\left (p_{i}^{(q)}\right)\!\min \left \{\pi \!\left (p_{i}^{*(q+1)}\!\right), \,\exp \left (\!h\left (p_{i}^{\phantom {\dot {i}\!}*(q+1)}\right)\!\right)\right \}}\!\right \}$, we take $p_{i}^{(q+1)}=p_{i}^{(q)}$; otherwise, we set $p_{i}^{(q+1)}=p_{i}^{\phantom {\dot {i}\!}*(q+1)}$.

## Dose-finding algorithm

In this section, we develop a two-stage Bayesian nonparametric adaptive dose-finding design based on a two-stage procedure and the above proposed hybrid algorithm. Let *c*_*e*_ and *c*_*d*_ be the threshold values for dose escalation and de-escalation, respectively. In our numerical illustration, *c*_*e*_ and *c*_*d*_ satisfying the restriction that *c*_*e*_+*c*_*d*_>1 can be selected by the data-dependent approach via simulation studies rather than some fixed values such that the trail has some desirable operating characteristics, for example, a relatively high accuracy index, which is defined in Equation (6.1) of Cheng (2011). For safety, we restrict the dose escalating or de-escalating for the next cohort of patients to only one dose level of change at a time. The two-stage Bayesian nonparametric adaptive dose-finding design is described as follows.

**(I) The start-up stage**

Patients in the first cohort are administered to the lowest dose level *d*_1_. If at least one toxicity is observed, the first stage is stopped and the second stage is conducted. If no toxicity is observed, patients in the second cohort are administered to the dose level *d*_2_. This process is continued until at least one toxicity is observed.

**(II) The second stage**

For dose level *d*_*k*_ administered to the *j*th cohort of patients, if there is at least one toxicity outcome observed, we denote $d_{k}^{(j)}$ as the dose level administered to the *j*th cohort of patients, i.e., the superscript *j* denotes the numerical order of cohort and the subscript *k* represents the dose level.

(i) Patients in the (*j*+1)th cohort are administered to dose level $d_{k}^{(j+1)}$. Based on the data of the first *j*+1 cohorts of patients, we can simultaneously obtain $\hat {\boldmath {p}}=(\hat {p}_{1},\ldots,\hat {p}_{\phantom {\dot {i}\!}S_{j+1}})$ and the toxicity probability $\text {Pr}(\hat {p}_{k}<\theta)$ at the current dose level $d_{k}^{(j+1)}$ via the above proposed hybrid algorithm.

(ii) If the probability $\text {Pr}(\hat {p}_{k}<\theta)>c_{e}$, the dose level administered to the (*j*+2)th cohort of patients is escalated to the dose level *d*_*k*+1_, i.e. $d_{k+1}^{(j+2)}=d_{k+1}$. If the current dose $d_{k}^{(j+1)}=d_{K}$, the dose administered to the (*j*+2)th cohort of patients is still *d*_*K*_, that is, $d_{K}^{(j+2)}=d_{K}$.

(iii) If the probability $\text {Pr}(\hat {p}_{k}<\theta)< c_{d}$, the dose level administered to the (*j*+2)th cohort of patients is deescalated to the dose level *d*_*k*−1_, i.e. $d_{k-1}^{(j+2)}=d_{k-1}$. If the current dose $d_{k}^{(j+1)}=d_{1}$, the dose level administered to the (*j*+2)th cohort of patients is still *d*_1_, that is, $d_{1}^{(j+2)}=d_{1}$.

(iv) Otherwise, the (*j*+2)th cohort of patients continues to be treated at the dose level *d*_*k*_ i.e., $d_{K}^{(j+2)}=d_{K}$.

(v) Once the maximum sample size *N* is attained, the dose level with the probability of toxicity being closest to *θ* is selected as the MTD.

## Simulation study

To investigate the finite sample performance of the nonparametric continual reassessment method (NCRM), several simulation studies are conducted for six toxicity scenarios together with eight dose levels (i.e., *K*=8), which are given in Table 1. Here, for simplicity, the dose levels are identified by a number from 1 to 8, that is, *d*_*k*_=*k* for *k*=1,…,8; and we take the target toxicity probability as *θ*=0.3. In Table 1, Scenario 1 indicates that the toxicity probability is steadily increasing and the target dose is level 5; Scenario 2 shows that the first five dose levels are the same as those given in Scenario 1, but the toxicity probability for dose level 6 suddenly jumps to an unacceptably high level 0.6; Scenario 3 has a flat relationship with the toxicity probability never attaining unacceptable levels, and the target dose is level 8; Scenario 4 has a flat relationship with the toxicity probability never reaching unacceptable levels, and there is no target dose but level 8 is quite close to the target dose given in Scenario 3; Scenario 5 implies that all the dose levels have an unacceptable toxicity probability, and the target dose is level 1; Scenario 6 is used to examine the situation that all the doses are over toxic, and there is no target dose but level 1 is quite close to the target dose. In practice, the trial would be stopped and doses will be reformulated once too many toxicities are occurred. We take sample size of each trial as *N*=60, the number of cohorts as *J*=20, and *m*=3. In implementing the Gibbs sampler, we collect 1000 observations after 700 burn-in iterations.

**Table 1:** Six toxicity scenarios for a single-agent trial with *θ*=0.3

		Dose level							
Scenario	Method	1	2	3	4	5	6	7	8
1	true	0.05	0.08	0.12	0.20	**0.30** ^**a**^	0.45	0.60	0.70
	skeleton	0.03	0.06	0.12	0.20	0.30	0.40	0.50	0.59
	*F*_0_	0.006	0.02	0.07	0.16	0.31	0.50	0.69	0.84
2	true	0.05	0.08	0.12	0.20	**0.30** ^**a**^	0.60	0.80	0.90
	skeleton	0.002	0.01	0.06	0.16	0.30	0.45	0.59	0.71
	*F*_0_	0.006	0.02	0.07	0.16	0.31	0.50	0.69	0.84
3	true	0.01	0.05	0.10	0.14	0.18	0.22	0.25	**0.30** ^**a**^
	skeleton	0.02	0.04	0.06	0.10	0.14	0.18	0.24	0.30
	*F*_0_	0.04	0.05	0.08	0.12	0.16	0.21	0.27	0.34
4	true	0.01	0.05	0.08	0.12	0.16	0.2	0.24	**0.26** ^**a**^
	skeleton	0.003	0.01	0.03	0.05	0.10	0.15	0.22	0.30
	*F*_0_	0.04	0.05	0.08	0.12	0.16	0.21	0.27	0.34
5	true	**0.30** ^**a**^	0.40	0.50	0.60	0.70	0.80	0.90	0.95
	skeleton	0.30	0.44	0.58	0.69	0.78	0.84	0.89	0.92
	*F*_0_	0.31	0.40	0.50	0.60	0.69	0.77	0.84	0.89
6	true	0.40	0.45	0.50	0.55	0.60	0.65	0.70	0.80
	skeleton	0.30	0.40	0.50	0.59	0.67	0.74	0.80	0.84
	*F*_0_	0.31	0.40	0.50	0.60	0.69	0.77	0.84	0.89

^a^Numbers in boldface are the target MTDs

For comparison, we consider two Bayesian model-based CRM dose-finding methods including the power model: $p_{i}=d_{i}^{\exp (\beta)}$ and one-parameter logistic model: *p*_*i*_= exp(*a*_0_+*βd*_*i*_)/{1+ exp(*a*_0_+*βd*_*i*_)} with *a*_0_=3, where the prior of *β* is assumed to follow the normal distribution with mean zero and variance 1.34, i.e., *β*∼*N*(0,1.34). For each of the above considered six scenarios, given *θ*, *k* and *K*, a good “skeleton” can be directly generated using the function ‘getprior’ in the R package with the optimal value of *δ*, which can be obtained by the algorithm of Lee and Cheung [17]. But, its computational burden is too expensive. To address this issue, we here use a fixed value, which is evaluated using the data-dependent approach via simulation studies from a prespecified indifference interval so that the trial has some desirable operating characteristics, for example, the prior expectation of *η* is close to its true values, to replace the optimal value of *δ*. For our considered six scenarios, simulation studies evidence that we can take *δ* as 0.05, 0.075, 0.03, 0.04, 0.07 and 0.05, respectively.

For each of the above considered six scenarios, we set the initial value *p*^(0)^ as its corresponding skeleton in implementing Gibbs sampler. When *α* and *F*_0_ are assumed to be known, to investigate the effect of the selection of *α*, we consider three cases of *α*: *α*=5, 10 and 20, corresponding to small, moderate and large values of *α*, respectively, and a standard normal distribution assumption for *F*_0_. As mentioned above, we choose appropriate values of *μ* and *σ* such that median of *F*_0_ should be consistent with the initial guess for the MTD. To wit, if there is a prior belief that dose level *d*_*k*_ is the MTD, we can select appropriate values of *μ* and *σ* such that $F_{0}(d_{k}|\eta)=\Phi \left (\frac {d_{k}-\mu }{\sigma }\right)=\theta $. Table 1 gives the values of *F*_0_’s corresponding to six scenarios together with eight dose levels, where *μ*=6 and *σ*=2 for scenario 1 and 2, *μ*=10 and *σ*=5 for scenario 3 and 4, and *μ*=3 and *σ*=4 for scenario 5 and 6, respectively.

The preceding proposed hybrid algorithm is used to calculate Bayesian estimates of *p*_*k*_’s, and the preceding proposed two-stage Bayesian nonparametric adaptive dose-finding algorithm is employed to determine MTD. To illustrate how the NCRM works, we present results of one simulation trial for scenario 1 together with *α*=5 in Table 2. Included in this table are: *i* (the serial number of the current experiment cohort); *x*_*i*_ (the dose administered to the current cohort of patients); *y*_*i*1_, *y*_*i*2_, *y*_*i*3_ (the observations for the current experiment cohort); $\hat {p}_{k}$ (estimate of toxicity probability for *k*=1,…,*K*). Examination of Table 2 shows that (i) dose level 5 (i.e., *d*_5_) is selected as the MTD and $\hat {p}_{5}=0.2977$, which is quite close to true toxicity probability 0.3; (ii) among 11 cohorts of patients administered to dose level *d*_5_, only 10 patients (i.e., $\sum \nolimits _{i=1}^{20}\sum \nolimits _{m=1}^{3}y_{{im}}$) are experienced toxicity; (iii) the doses with the relatively high toxicity probability (such as *d*_7_ and *d*_8_) may have no chance to be administered to patients, which guarantees the safety of patients.

**Table 2:** Bayesian estimates of *p*_*k*_’s via NCRM under scenario 1

True					0.05	0.08	0.12	0.20	0.30	0.45	0.60	0.70
*i*	*x*_*i*_	*y*_*i*1_	*y*_*i*2_	*y*_*i*3_	$\hat {p}_{1}$	$\hat {p}_{2}$	$\hat {p}_{3}$	$\hat {p}_{4}$	$\hat {p}_{5}$	$\hat {p}_{6}$	$\hat {p}_{7}$	$\hat {p}_{8}$
1	*d*_1_	0	0	1								
2	*d*_1_	0	0	0	0.0156	-	-	-	-	-	-	-
3	*d*_2_	0	0	0	0.0278	0.0380	-	-	-	-	-	-
4	*d*_3_	1	0	0	0.0439	0.0573	0.0789	-	-	-	-	-
5	*d*_4_	0	0	0	0.0501	0.0703	0.1014	0.1336	-	-	-	-
6	*d*_5_	0	0	0	0.0585	0.0809	0.1169	0.1534	0.2109	-	-	-
7	*d*_6_	0	0	1	0.0589	0.0852	0.1248	0.1682	0.2377	0.3426	-	-
8	*d*_5_	0	1	0	0.0595	0.0734	0.1196	0.1672	0.2430	0.3457	-	-
9	*d*_6_	1	1	1	0.0711	0.1018	0.1496	0.2006	0.2841	0.4060	-	-
10	*d*_5_	0	1	0	0.0763	0.0954	0.1484	0.2023	0.2864	0.4063	-	-
11	*d*_5_	0	1	1	0.0681	0.0995	0.1528	0.2112	0.3116	0.4156	-	-
12	*d*_5_	0	0	1	0.0814	0.1144	0.1668	0.2227	0.3190	0.4196	-	-
13	*d*_4_	0	0	0	0.0702	0.0979	0.1474	0.1965	0.3072	0.4144	-	-
14	*d*_5_	0	0	1	0.0670	0.0979	0.1431	0.1958	0.3104	0.4151	-	-
15	*d*_5_	0	0	0	0.0672	0.0960	0.1421	0.1915	0.2904	0.4062	-	-
16	*d*_5_	0	0	1	0.0767	0.1073	0.1549	0.2026	0.2989	0.4094	-	-
17	*d*_5_	0	1	0	0.0704	0.1004	0.1450	0.1941	0.2958	0.4070	-	-
18	*d*_5_	1	1	0	0.0723	0.1039	0.1546	0.2066	0.3196	0.4205	-	-
19	*d*_4_	0	0	0	0.0632	0.0916	0.1369	0.1842	0.3128	0.4170	-	-
20	*d*_5_	0	0	0	0.0730	0.1046	0.1428	0.1868	0.2977	0.4103	-	-

**Fig. 1:**
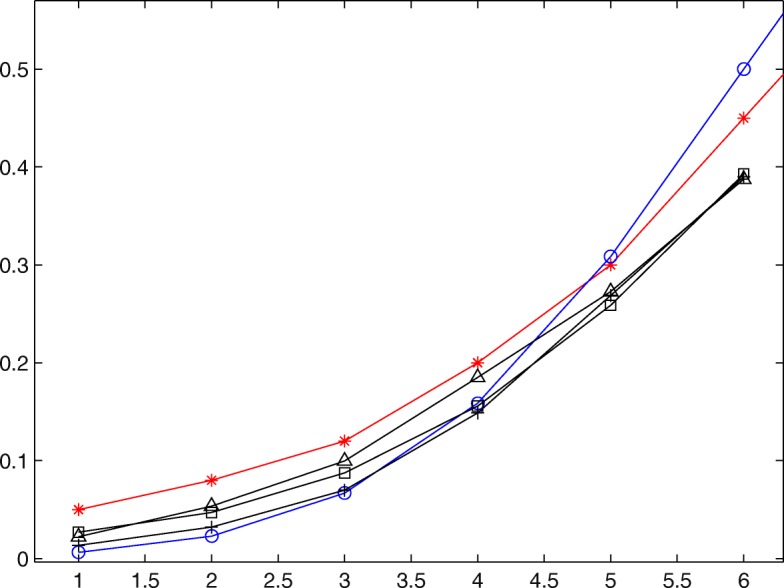
Nonparametric Bayesian estimation of dose-toxicity curve under scenarios 1 when *α* and *F*_0_ are known

**Fig. 2:**
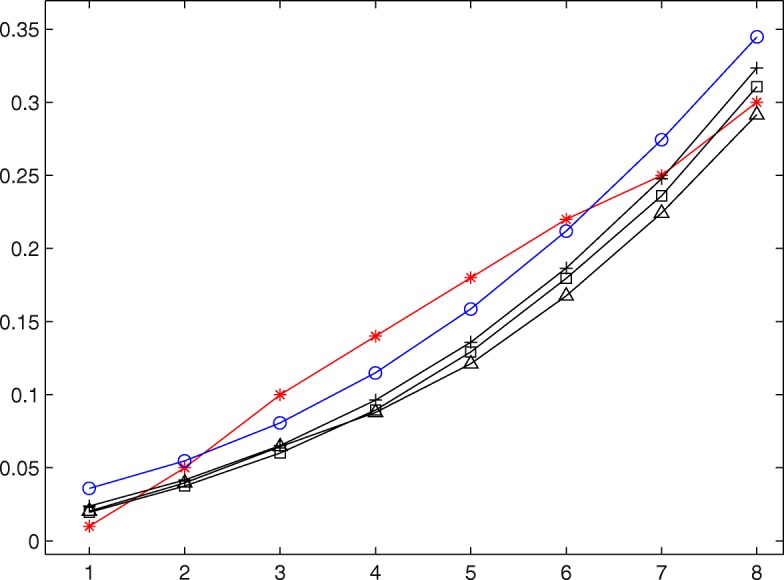
Nonparametric Bayesian estimation of dose-toxicity curve under scenarios 3 when *α* and *F*_0_ are known

Figures 1 and 2 plot Bayesian nonparametric estimations of unknown dose-toxicity curve for three specified values of *α* for scenarios 1 and 3, respectively. In each figure, “ −⋆” represents the dose-toxicity curve corresponding to true dose-toxicity data, “ −∘” corresponds to the base curve, and “$-\vartriangle $”, “$-\square $” and “-+” correspond to the estimated dose-toxicity curves for *α*=5, 10 and 20, respectively. Examination of Figs. 1 and 2 show that the estimated curves are more and more close to *F*_0_ with the increase of *α* when the doses administered to patients are less than but close to the target dose, which is consistent with the conclusion that the large value of *α* reflects a prior belief that *F* is tight around *F*_0_.

For the aforementioned NCRM, we calculate the selection probabilities that a dose level is selected as the MTD, the total numbers of toxicities observed, and average numbers of patients that are administered to each of eight dose levels for the above specified six scenarios together with *α*=5 and 20. For comparison, we also calculate the results corresponding to logistic model and power model. Results for 1000 simulated trials are given in Tables 3 and 4. Examination of Tables 3 and 4 show that the above developed Bayesian NCRM performs better than two parametric CRMs (i.e., logistic and power models) in terms of the following four aspects: (i) Bayesian NCRM generally selects the MTD with a relatively higher probability than two parametric CRMs; (ii) Bayesian NCRM consistently selects over-MTD with a relatively lower probability than two parametric CRMs; (iii) the total number of toxicities observed are almost identical for all three methods under our considered cases; (iv) Bayesian NCRM has a higher percentage of patients treated at MTD than two parametric CRMs, except for scenario 3 with *α*=5, but Bayesian NCRM treats more patients at dose levels below MTD and less patients at dose levels above MTD than two parametric CRMs; (v) the selection probabilities for scenarios 3 and 4 are smaller than those for other four scenarios because the locations of target doses for scenarios 3 and 4 are different from those for other scenarios, which indicates that the highest dose should be carefully administered to patients for safety, and patients should be administered to the lower dose level.

**Table 3:** Selection probabilities and total numbers of toxicities observed for logistic model, power model and NCRM under six scenarios

		Dose level								# of	# of
Scenario	Method	1	2	3	4	5	6	7	8	Tox.	Pat.
1	Logistic	0.000	0.000	0.008	0.227	0.637	0.128	0.000	0.000	15	60
	Power	0.000	0.000	0.004	0.212	0.664	0.120	0.000	0.000	15	60
	NCRM5^a^	0.000	0.002	0.001	0.161	0.773	0.063	0.000	0.000	15	60
	NCRM2^a^	0.000	0.000	0.000	0.007	0.980	0.013	0.000	0.000	16	60
2	Logistic	0.000	0.001	0.042	0.362	0.571	0.024	0.000	0.000	13	60
	Power	0.000	0.001	0.028	0.369	0.581	0.021	0.000	0.000	14	60
	NCRM5	0.002	0.000	0.005	0.292	0.698	0.003	0.000	0.000	15	60
	NCRM2	0.000	0.000	0.000	0.186	0.814	0.000	0.000	0.000	14	60
3	Logistic	0.000	0.000	0.000	0.002	0.043	0.108	0.306	0.541	13	60
	Power	0.000	0.000	0.000	0.001	0.031	0.104	0.311	0.553	13	60
	NCRM5	0.000	0.001	0.007	0.019	0.028	0.117	0.373	0.455	13	60
	NCRM2	0.000	0.000	0.000	0.000	0.000	0.008	0.379	0.613	13	60
4	Logistic	0.000	0.000	0.000	0.002	0.036	0.143	0.286	0.533	11	60
	Power	0.000	0.000	0.000	0.000	0.028	0.128	0.321	0.523	11	60
	NCRM5	0.000	0.001	0.003	0.007	0.011	0.086	0.289	0.603	12	60
	NCRM2	0.000	0.000	0.000	0.000	0.000	0.004	0.263	0.733	12	60
5	Logistic	0.804	0.189	0.007	0.000	0.000	0.000	0.000	0.000	20	60
	Power	0.811	0.183	0.006	0.000	0.000	0.000	0.000	0.000	20	60
	NCRM5	0.898	0.100	0.002	0.000	0.000	0.000	0.000	0.000	19	60
	NCRM2	0.971	0.029	0.000	0.000	0.000	0.000	0.000	0.000	19	60
6	Logistic	0.970	0.027	0.003	0.000	0.000	0.000	0.000	0.000	24	60
	Power	0.972	0.028	0.000	0.000	0.000	0.000	0.000	0.000	24	60
	NCRM5	0.987	0.013	0.000	0.000	0.000	0.000	0.000	0.000	24	60
	NCRM2	1.000	0.000	0.000	0.000	0.000	0.000	0.000	0.000	24	60

^a^NCRM5 and NCRM2 denote NCRM method with *α*=5 and 20, respectively

**Table 4:** Average numbers of patients treated at each of eights doses for logistic model, power model and NCRM

		Dose level							
Scenario	Method	1	2	3	4	5	6	7	8
1	Logistic	3.579	4.008	5.916	14.463	23.304	8.124	0.576	0.030
	Power	3.489	3.954	5.622	14.739	23.889	7.755	0.543	0.009
	NCRM5^a^	3.474	3.615	3.912	15.462	27.387	6.009	0.141	0.000
	NCRM2^a^	3.423	3.558	3.708	8.298	35.454	5.493	0.066	0.000
2	Logistic	3.582	4.632	8.868	19.206	21.147	2.535	0.030	0.000
	Power	3.486	4.158	8.004	20.496	21.663	2.178	0.015	0.000
	NCRM5	3.447	3.648	4.569	18.543	25.698	4.065	0.300	0.000
	NCRM2	3.447	3.513	3.693	19.977	27.825	1.497	0.048	0.000
3	Logistic	3.108	3.486	4.137	5.274	6.882	9.096	11.724	16.293
	Power	3.066	3.480	4.089	5.157	6.552	8.970	12.342	16.344
	NCRM5	3.090	3.435	3.684	3.897	4.908	8.901	17.457	14.628
	NCRM2	3.057	3.420	3.660	3.636	3.531	3.930	21.168	17.598
4	Logistic	3.105	3.483	3.942	4.977	7.266	10.125	11.610	15.492
	Power	3.066	3.468	3.843	4.737	6.573	10.266	12.861	15.186
	NCRM5	3.135	3.366	3.453	3.777	4.395	7.809	16.281	17.784
	NCRM2	3.084	3.387	3.573	3.612	3.522	3.630	18.879	20.313
5	Logistic	44.637	12.972	2.229	0.156	0.006	0.000	0.000	0.000
	Power	45.189	12.822	1.848	0.135	0.006	0.000	0.000	0.000
	NCRM5	49.740	8.958	1.176	0.114	0.012	0.000	0.000	0.000
	NCRM2	53.178	6.366	0.408	0.042	0.006	0.000	0.000	0.000
6	Logistic	54.468	4.467	0.894	0.141	0.030	0.000	0.000	0.000
	Power	54.573	4.500	0.810	0.102	0.015	0.000	0.000	0.000
	NCRM5	57.676	2.865	0.414	0.039	0.006	0.000	0.000	0.000
	NCRM2	58.149	1.596	0.237	0.018	0.000	0.000	0.000	0.000

^a^NCRM5 and NCRM2 denote NCRM method with *α*=5 and 20, respectively

Now we assume that *α* and *F*_0_ are unknown. As an illustration of the above presented Bayesian NCRM procedure, here we only consider scenarios 1 and 3. For scenario 1, we consider four different priors on *α*: *Γ*(2,5), *Γ*(2,2), *Γ*(5,2) and *Γ*(10,2), which correspond to small and moderate expectations of *α*, and four discrete uniform priors for (*μ,σ*) on the rectangles (5,7)×(1,3), (4,8)×(1,3), (5,7)×(0,4) and (4,8)×(0,4), which indicate that prior expectations of *μ* and *σ* are 6 and 2, respectively. For scenario 3, we consider the same priors on *α* as scenario 1, but the following four different discrete uniform priors of (*μ,σ*) on the rectangles (9,11)×(4,6), (8,12)×(4,6), (9,11)×(3,7) and (8,12)×(3,7), which imply that prior expectations of *μ* and *σ* are 10 and 5, respectively.

Based on the above considered settings, the preceding introduced hybrid algorithm is adopted to evaluate Bayesian estimation of *p*_*k*_’s, and the preceding developed two-stage Bayesian nonparametric adaptive dose-finding algorithm is employed to determine the MTD. Similarly, we also calculate the selection probabilities, total numbers of toxicities observed and average numbers of patients treated at each of eight dose levels for scenarios 1 and 3. Results for 1000 simulated trials are given in Tables 5 and 6. Examination of Table 5 and 6 shows that (i) the selection probabilities and the number of patients treated at the MTD increase with the increase of prior expectation of *α*; (ii) the selection probabilities and the number of patients treated at the dose level closet to the MTD decrease with the increase of prior expectation of *α* for scenario 1, but increase with the increase of prior expectation of *α* for scenario 3; (iii) the total number of toxicities observed are almost equal regardless of the priors of *α* and (*μ,σ*), which shows that there is little effect of the selection of the priors of *α*, *μ* and *σ* on the total number of toxicities observed.

**Table 5:** Selection probabilities and total numbers of toxicities observed for NCRM under scenarios 1 and 3 when *α* and (*μ,σ*) are unknown

Prior			Dose Level								# of	# of
Case	(*μ,σ*)^a^	*α*	1	2	3	4	5	6	7	8	Tox.	Pat.
1	A	*Γ*(2,5)	0.012	0.016	0.016	0.200	0.646	0.110	0.000	0.000	15	60
		*Γ*(2,2)	0.002	0.001	0.001	0.167	0.771	0.057	0.001	0.000	15	60
		*Γ*(5,2)	0.000	0.001	0.001	0.092	0.853	0.053	0.000	0.000	15	60
		*Γ*(10,2)	0.000	0.000	0.000	0.064	0.896	0.040	0.000	0.000	16	60
	B	*Γ*(2,5)	0.009	0.008	0.015	0.236	0.647	0.081	0.004	0.000	14	60
		*Γ*(2,2)	0.000	0.000	0.000	0.139	0.817	0.044	0.000	0.000	15	60
		*Γ*(5,2)	0.000	0.000	0.000	0.125	0.826	0.049	0.000	0.000	15	60
		*Γ*(10,2)	0.000	0.000	0.000	0.092	0.861	0.047	0.000	0.000	15	60
	C	*Γ*(2,5)	0.000	0.000	0.002	0.172	0.776	0.050	0.000	0.000	15	60
		*Γ*(2,2)	0.000	0.000	0.000	0.162	0.783	0.055	0.000	0.000	15	60
		*Γ*(5,2)	0.000	0.000	0.001	0.107	0.831	0.061	0.000	0.000	15	60
		*Γ*(10,2)	0.000	0.000	0.000	0.064	0.881	0.055	0.000	0.000	16	60
	D	*Γ*(2,5)	0.000	0.000	0.004	0.219	0.726	0.050	0.001	0.000	15	60
		*Γ*(2,2)	0.000	0.000	0.001	0.177	0.783	0.039	0.000	0.000	15	60
		*Γ*(5,2)	0.000	0.000	0.001	0.152	0.796	0.051	0.000	0.000	15	60
		*Γ*(10,2)	0.000	0.000	0.000	0.129	0.834	0.037	0.000	0.000	15	60
3	E	*Γ*(2,5)	0.001	0.005	0.015	0.048	0.094	0.157	0.242	0.438	13	60
		*Γ*(2,2)	0.001	0.005	0.008	0.032	0.059	0.138	0.294	0.463	12	60
		*Γ*(5,2)	0.001	0.004	0.009	0.013	0.026	0.095	0.377	0.475	13	60
		*Γ*(10,2)	0.000	0.000	0.000	0.000	0.000	0.020	0.400	0.580	13	60
	F	*Γ*(2,5)	0.001	0.007	0.018	0.041	0.077	0.162	0.239	0.455	13	60
		*Γ*(2,2)	0.001	0.004	0.013	0.017	0.044	0.118	0.347	0.456	13	60
		*Γ*(5,2)	0.000	0.001	0.001	0.000	0.008	0.059	0.410	0.521	13	60
		*Γ*(10,2)	0.000	0.000	0.000	0.000	0.000	0.046	0.425	0.529	13	60
	G	*Γ*(2,5)	0.003	0.009	0.015	0.039	0.083	0.145	0.258	0.448	13	60
		*Γ*(2,2)	0.000	0.004	0.017	0.031	0.048	0.127	0.318	0.455	13	60
		*Γ*(5,2)	0.001	0.002	0.004	0.004	0.017	0.071	0.376	0.525	13	60
		*Γ*(10,2)	0.000	0.001	0.000	0.000	0.000	0.014	0.357	0.628	13	60
	H	*Γ*(2,5)	0.001	0.001	0.017	0.032	0.073	0.144	0.264	0.468	12	60
		*Γ*(2,2)	0.001	0.005	0.005	0.014	0.019	0.089	0.350	0.517	13	60
		*Γ*(5,2)	0.000	0.001	0.001	0.002	0.006	0.064	0.364	0.562	13	60
		*Γ*(10,2)	0.000	0.000	0.000	0.000	0.000	0.024	0.406	0.570	13	60

^a^Note: *A*=(5,7)×(1,3), *B*=(4,8)×(1,3), *C*=(5,7)×(0,4), *D*=(4,8)×(0,4), *E*=(9,11)×(4,6), *F*=(8,12)×(4,6), *G*=(9,11)×(3,7), *H*=(8,12)×(3,7)

**Table 6:** Average numbers of patients treated at each of eight doses for NCRM under scenario 1 and 3 when *α* and (*μ,σ*) are unknown

Prior			Dose Level							
Case	(*μ,σ*)^a^	*α*	1	2	3	4	5	6	7	8
1	A	*Γ*(2,5)	3.474	3.915	6.273	15.879	21.567	7.983	0.879	0.030
		*Γ*(2,2)	3.342	3.606	4.263	15.705	26.751	6.030	0.303	0.000
		*Γ*(5,2)	3.390	3.672	3.705	12.576	30.048	6.426	0.177	0.006
		*Γ*(10,2)	3.450	3.603	3.675	10.635	31.983	6.582	0.066	0.006
	B	*Γ*(2,5)	3.537	3.957	6.381	17.718	21.378	6.450	0.555	0.024
		*Γ*(2,2)	3.342	3.618	3.810	15.129	28.473	5.367	0.165	0.006
		*Γ*(5,2)	3.393	3.510	3.702	14.523	29.253	5.460	0.159	0.000
		*Γ*(10,2)	3.363	3.594	3.717	13.014	31.074	5.157	0.075	0.006
	C	*Γ*(2,5)	3.417	3.570	4.155	14.847	26.706	7.101	0.204	0.00
		*Γ*(2,2)	3.411	3.573	3.948	14.589	28.089	6.339	0.051	0.000
		*Γ*(5,2)	3.372	3.570	3.783	12.495	29.781	6.819	0.180	0.000
		*Γ*(10,2)	3.402	3.567	3.663	10.887	31.743	6.621	0.105	0.012
	D	*Γ*(2,5)	3.435	3.525	4.404	18.249	25.404	4.761	0.210	0.012
		*Γ*(2,2)	3.444	3.558	3.927	16.455	28.017	4.455	0.144	0.000
		*Γ*(5,2)	3.429	3.501	3.798	16.017	28.530	4.566	0.153	0.006
		*Γ*(10,2)	3.408	3.612	3.684	15.390	29.757	4.053	0.090	0.006
3	E	*Γ*(2,5)	3.075	3.399	3.687	4.335	6.141	9.936	15.465	13.962
		*Γ*(2,2)	3.108	3.486	3.714	4.305	5.871	9.753	16.014	13.749
		*Γ*(5,2)	3.078	3.363	3.810	3.870	4.554	8.478	18.222	14.625
		*Γ*(10,2)	3.090	3.396	3.633	3.684	3.576	4.617	21.420	16.584
	F	*Γ*(2,5)	3.090	3.471	3.741	4.326	6.333	10.383	15.444	13.212
		*Γ*(2,2)	3.111	3.375	3.735	4.113	5.418	9.525	17.049	13.674
		*Γ*(5,2)	3.081	3.450	3.633	3.645	3.801	6.630	19.911	15.849
		*Γ*(10,2)	3.105	3.387	3.657	3.708	3.588	5.532	21.4189	15.834
	G	*Γ*(2,5)	3.096	3.477	3.789	4.275	6.015	9.840	15.312	14.196
		*Γ*(2,2)	3.096	3.450	3.651	4.245	5.706	9.441	16.191	14.220
		*Γ*(5,2)	3.372	3.375	3.792	3.735	4.005	6.873	19.104	16.056
		*Γ*(10,2)	3.066	3.474	3.681	3.597	3.570	4.347	20.088	18.177
	H	*Γ*(2,5)	3.060	3.411	3.753	4.272	5.952	9.834	15.462	14.256
		*Γ*(2,2)	3.081	3.426	3.672	3.849	4.434	7.320	18.411	15.807
		*Γ*(5,2)	3.051	3.477	3.648	3.672	3.747	6.306	19.278	16.821
		*Γ*(10,2)	3.075	3.396	3.666	3.648	3.633	4.785	20.613	17.184

^a^Note: *A*=(5,7)×(1,3), *B*=(4,8)×(1,3), *C*=(5,7)×(0,4), *D*=(4,8)×(0,4), *E*=(9,11)×(4,6), *F*=(8,12)×(4,6), *G*=(9,11)×(3,7), *H*=(8,12)×(3,7)

## Results

According to the above presented simulation study, we have the following results. First, the estimated MTD in single-agent dose-finding clinical trials via our proposed two-stage Bayesian nonparametric adaptive dose-finding algorithm is quite close to true toxicity probability under our considered settings. Second, the doses with the relatively high toxicity probability may have no chance to be assigned to patients, which guarantees the safety of patients. Third, the value of the weight *α* in the DP prior is a measure of a prior belief on the base distribution. Fourth, when the parameters in the DP prior are fixed, the proposed Bayesian NCRM behaves better than traditional model-based CRM; but when the parameters in the DP prior are unknown, the selection of the weight *α* has a positive effect on the selection probabilities and the number of patients treated at the MTD, while the selection of parameters in the DP prior has little effect on the total number of toxicities observed. In a word, numerical results show the flexibility of the proposed method for single-agent dose-finding trials, and the proposed method outperforms two classical CRMs under our considered scenarios.

## Discussion

Although this manuscript only considers a single agent dose-finding design, the proposed Bayesian NCRM can be extended to two-agent dose-finding studies, which is our further research topic. On the other hand, this paper only considers the evaluation of the toxicity of novel drug treatment, i.e., phase I clinical trial, but the developed Bayesian NCRM procedure can be extended to Bayesian nonparametric phase I/II dose-finding trial design that simultaneously allows for toxicity and efficiency of novel drug treatment for precision medicine.

## Conclusions

This paper proposes a Bayesian nonparametric continual reassessment method to estimate the MTD for a single-agent in phase I clinical trials. We relax the traditional parametric model assumption imposed on dose-toxicity relationship using a DP prior to approximate unknown distribution of dose-toxicity curve. A Bayesian method is developed to estimate toxicity probabilities of dose levels considered. A hybrid algorithm combining the Gibbs sampler and adaptive rejection Metropolis sampling algorithm is developed to generate observations from joint conditional distributions required in evaluating Bayesian estimates of toxicity probabilities of dose levels. A two-stage Bayesian nonparametric adaptive dose-finding design is developed to estimate the MTD. In the proposed Bayesian nonparametric adaptive dose-finding design, the dose administered to next cohort of patients can be escalated or deescalated to adjacent dose level more safety; generally, doses with a relatively higher toxicity probability may have no chance to be administered to patients, which guarantees the safety of patients. Simulation studies evidence that the proposed dose-finding procedure is model-free and robust, and performs better than two parametric models even in small sample sizes.

## Abbreviations

ARMS: Adaptive rejection Metropolis sampling
CRM: Continual reassessment method
DP: Dirichlet process
DLT: Dose-limiting toxicity
MTD: Maximum tolerated dose
NCRM: Nonparametric Continual reassessment method

## References

[1] O’Quigley J, Pepe M, Fisher L (1990). Continual reassessment method: a practical design for phase 1 clinical trials in cancer. Biometrics.

[2] Whitehead J, Brunier H (1995). Bayesian decision procedures for dose determining experiments. Stat Med.

[3] Piantadosi S, Fisher JD, Grossman S (1998). Practical implementation of a modified continual reassessment method for dose-finding trials. Cancer Chemother Pharmacol.

[4] Heyd JM, Carlin BP (1999). Adaptive design improvements in the continual reassessment method for phase I studies. Stat Med.

[5] Leung DH, Wang YG (2002). An extension of the continual reassessment method using decision theory. Stat Med.

[6] Yuan Z, Chappell R, Bailey H (2007). The Continual Reassessment Method for Multiple Toxicity Grades: A Bayesian Quasi ikelihood Approach. Biometrics.

[7] Yin G, Yuan Y (2009). Bayesian Model Averaging Continual Reassessment Method in Phase I Clinical Trials. J Am Stat Assoc.

[8] Møller S (2010). An extension of the continual reassessment methods using a preliminary up-and-down design in a dose finding study in cancer patients, in order to investigate a greater range of doses. Stat Med.

[9] Fan SK, Lu Y, Wang YG (2012). A simple Bayesian decision-theoretic design for dose-finding trials. Stat Med.

[10] Morita S, Thall PF, Takeda K (2017). A simulation study of methods for selecting subgroup-specific dosesin phase i trials. Pharm Stat.

[11] Gelfand AE, Kuo L (1991). Nonparametric bayesian bioassay including ordered polytomous response. Biometrika.

[12] Mukhopadhyay S (2000). Bayesian nonparametric inference on the dose level with specified response rate. Biometrics.

[13] Gasparini M, Eisele J (2000). A curve-free method for phase i clinical trials. Biometrics.

[14] Cheng YK (2011). Dose Finding by the Continual Reassessment Method.

[15] Ivanova A, Wang K (2006). Bivariate isotonic design for dose-finding with ordered groups. Stat Med.

[16] Yan F, Mandrekar SJ, Yuan Y (2017). Keyboard: a novel bayesian toxicity probability interval design for phase i clinical trials. Clin Cancer Res Off J Am Assoc Cancer Res.

[17] Lee SM, Cheung YK (2009). Modal calibration in the continual reassessment method. Clin Trials.

[18] Ramsey FL (1972). A Bayesian approach to bioassay. Biometrics.

[19] Escobar MD, West M (1995). Bayesian density estimation and inference using mixtures. Publ Am Stat Assoc.

[20] West M (1997). Hierarchial mixture models in neurological transmission analysis. J Am Stat Assoc.

